# The Equilibria of Triterpene Sapogenins–Phosphatidylcholine in Monolayers at the Air/Water Interface

**DOI:** 10.3390/ijms242216144

**Published:** 2023-11-09

**Authors:** Katarzyna Karwowska, Wiesław Urbaniak, Aneta D. Petelska

**Affiliations:** 1 Faculty of Chemistry, University of Bialystok, K. Ciolkowskiego 1K, 15-245 Bialystok, Poland; k.karwowska@uwb.edu.pl; 2 Faculty of Mechatronics, Kazimierz Wielki University, Chodkiewicz 30, 85-867 Bydgoszcz, Poland; wurban@ukw.edu.pl

**Keywords:** oleanolic acid, asiatic acid, 1,2-Dipalmitoyl-sn-glycero-3-phosphocholine (DPPC), monolayer, Langmuir method, BAM method, complex formation

## Abstract

Sapogenins are the non-sugar parts of saponins (aglycones), high-molecular-weight glycosides linked to one or more sugar side chains. This group of compounds presents many properties, e.g., the potent properties of reducing surface tension and foaming properties, as evidenced by the amphipathic nature of these substances. They are used in the cosmetics industry, the washing and detergent industry, and the food industry. In addition, they have many healing properties. They lower blood cholesterol but are also used to synthesize steroid drugs or hormones. As reported in the literature, saponins also show antitumor activity, leading to cell cycle inhibition and apoptosis of various neoplastic cells. In this study, the influence of two sapogenins: asiatic acid (AA) and oleanolic acid (OA), on the properties of monolayers made of phosphatidylcholine (DPPC) was investigated. The method used in these studies was the Langmuir method with Brewster angle microscopy. The interactions between the tested compounds in mixed monolayers were described. Using mathematical equations, we established that oleanolic acid and asiatic acid formed complexes with DPPC at 1:1 ratios, characterized by high stability constants. We derived the parameters characterizing the formed complexes and described the phase transitions that occur during the formation of pure and mixed monolayers.

## 1. Introduction

Saponins are a large group of organic compounds belonging to glycosides with many properties and applications. The source of these unusual substances is mainly plants, but their presence has also been noted in some marine organisms, such as sea cucumbers or starfish [1,2,3,4]. One of the types of saponins constitutes triterpene saponins, which are the most widely distributed in the plant world. They characterize specific pentacyclic construction. Triterpenoid saponins comprise a non-saccharide aglycone (genin or sapogenin) and a sugar part. They usually contain an α-amarin aglycone with 30 carbon atoms, unlike steroid saponins with a sterane skeleton of 27 carbon atoms. Triterpenoid saponins generally have four oxygen atoms: in the hydroxyl group (-OH) in the C-3 position, in the carboxyl group (-COOH) in the C-28 position, and the last oxygen atom presents as a non-attached alcoholic group (-CH2-OH) at C-24 [5,6]. The sugar portion of the saponins, called the glycone, is usually oligosaccharides. Oligosaccharides can be linked to a sapogenin via an ether or ester linkage at one or two glycosylation sites to give monodesmoside or bidesmoside saponins, respectively. Saponins with three saccharide chains are rare [7]. Saponins present many biological and pharmacological activities, mainly anti-inflammatory, molluscicidal, antiviral, antifungal, hypoglycemic, and hypocholesterolemic. Thanks to many properties, such as foaming, emulsifying, and hemolytic properties, saponins have been used in the washing industry, beverages, confectionery, cosmetics, and pharmaceutical products. They are often the main ingredients of many herbal medicines and have numerous pharmacological properties [8,9].

**Figure 1:**
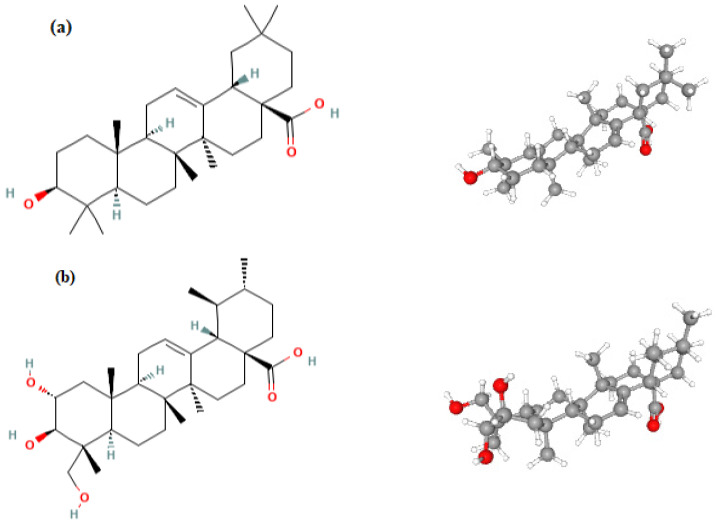
Structure of oleanolic acid (**a**) and asiatic acid (**b**).

The figures below show the structure of two compounds from the group of triterpene sapogenins, which are part of the saponins. Oleanolic acid (Figure 1a) is a natural compound in food and medicinal plants. A high concentration of this compound is shown, for example, in vegetable oils [10], fruits [11], andherbs such as lemon balm [12]. Oleanolic acid is also isolated from medicinal plants. Its sources are leaves [13,14], flowers, or roots [15,16]. Especially rich in oleanolic acid are olives and the olive tree. This compound is isolated from olives, virgin olive oil, and leaves of the olive tree [17,18]. Asiatic acid (Figure 1b) is also a triterpene sapogenin. It is the main ingredient in some medicinal herbs and is also widely present in all parts of various plants [19]. The sapogenin is also the main ingredient in some medicinal herbs and is also widely present in all aspects of different plants [20]. Its sources are, e.g., basil (*Ocimumbasilicum*) [21] andcentella (*Centella asiatica* L.) [22]. Both oleanolic acid and asiatic acid have many valuable properties. Particular attention is given to the use of these compounds in medicine. These sapogenins have been reported to possess antioxidative [23], hepatoprotective, anti-inflammatory [24], and anticancer activities [25]. The antitumor property is considered one of the most important due to the increasing number of oncological patients. It turns out that these sapogenins have a cytotoxic effect on various cancer cells. They inhibit the proliferation of cancer cells [26] and cause apoptosis in many tumor cells, e.g., lung cancer cells, liver cancer cells [27], prostate cancer cells, andbreast carcinoma cells [28].

The examinations presented in this paper are a continuation of our research on the interaction of model membranes (1,2-dipalmitoyl-sn-glycero-3-phosphocholine (DPPC)) and molecules of increasing complexity (oleanolic acid and asiatic acid). In this paper, we analyze the effect ofthe triterpene sapogenins (oleanolic acid and asiatic acid) on the model lipid membrane formed of the 1,2-dipalmitoyl-sn-glycero-3-phosphocholine in (DPPC)-AA and DPPC-OA systems. We study the changes in surface pressure during compression of the monolayer and the area of a single molecule in the model monolayer—DPPC-TS. We also compare the complexes’ parameters (stability constants and the surface areas).

Since these parameters influence the interactions between the membranes and biologically active compounds—two triterpene sapogenins (OA and AA)—the data presented below may help one to understand the membrane component’s binding mechanisms. Our study is aimed at the theoretical and visual description of the built mixed monolayer structures.

### 1.1. Theory

Triterpene sapogenin (TS) and 1,2-dipalmitoyl-sn-glycero-3-phosphocholine (DPPC) can form complexes in various weight ratios, but the complex with a stoichiometry of 1:1 is considered to be the dominant one. We, therefore, assumed that this was correct because, in complexes, the first stability constant is usually the largest [29].

The complexation equilibrium (1:1 complex) is presented below:

(1) DPPC+TS ↔DPPC−TS

The equilibrium state of the complex might be described by the system of equations [30,31]:

(2) $$a_{DPPC}S_{DPPC}+a_{TS}S_{TS}+a_{DPPC-TS}S_{DPPC-TS}=1$$

(3) $$a_{DPPC}+a_{DPPC-TS}=c_{DPPC}$$

(4) $$a_{TS}+a_{DPPC-TS}=c_{TS}$$

(5) $$K_{DPPC-TS}=\frac{a_{DPPC-TS}}{a_{DPPC}\;\cdot \;a_{TS}}$$

(6) $$x_{TS}=\frac{C_{TS}}{c_{DPPC}+c_{TS}}$$

where $a_{DPPC}$, $a_{TS}$, and $a_{DPPC-TS}$ (mol m^−2^) are the surface concentrations of components: DPPC and TS and complex DPPC-TS; $c_{DPPC}$ and $c_{TS}$ (mol m^−2^) are the total surface concentrations of components: DPPC and TS; $S_{DPPC}$,${\;S}_{TS}$, and $S_{DPPC-TS}$ (m^2^ mol^−1^) are the surface areas occupied by 1 mole of components: DPPC and TS and complex DPPC-TS; $K_{DPPC-TS}$ (m^2^ mol^−1^) is the stability constant of complex DPPC-TS; and $x_{DPPC}$ and $x_{TS}$ are the mole fractions of components DPPC and TS.

The system of Equations (2)–(5) contains unknown quantities $a_{DPPC}$, $a_{TS}$, $a_{DPPC-TS}$, $S_{DPPC-TS}$, and $K_{DPPC-TS}$ as well as known or easy-to-determine quantities $S_{DPPC}$, $S_{TS}$, $x_{TS}$, $c_{DPPC}$, and $c_{TS}$.

Attempts to solve this system of equations resulted in complicated expressions, so Equations (2)–(5) were differentiated concerning and approximated to low or high argument values. Such considerations led to the simplification of the system of Equations (2)–(5) which was presented in earlier papers [30,31].

The final equations are [30,31]:

- The stability constant of the complex $K_{DPPC-TS}$ calculation:
  (7) $$K_{DPPC-TS}=\frac{S_{TS}^{3}{\;c}_{TS\;(x_{TS}=1)}^{\prime }-2\;S_{DPPC\;}S_{TS\;}-S_{DPPC}^{3}{\;c}_{DPPC(x_{TS}=0)}}{S_{TS}-S_{DPPC}+S_{DPPC}^{2}{\;c}_{DPPC\left(x_{TS}=0\right)}^{\prime }+S_{TS}^{2}c_{TS(x_{TS}=1)}^{\prime }}$$
- The surface area occupied by one molecule of the complex $S_{DPPC-TS}$ calculation:
  (8) $$S_{DPPC-TS}=\frac{\left(S_{DPPC}{\;S}_{TS}+c_{DPPC\;\left(x_{TS}=0\right)}^{\prime }c_{TS\;\left(x_{TS}=1\right)}^{\prime }S_{DPPC\;}^{2}S_{TS}^{2})(S_{DPPC}+S_{TS}\right)}{S_{DPPC}^{3}c_{DPPC(x_{TS}=0)}^{\prime }+S_{TS}^{3}{\;c}_{TS(x_{TS}=1)}^{\prime }}$$

The slopes of tangent lines at the points(pure component DPPC) $x_{TS}=0$ and $x_{TS}=1$ (pure component TS) may be calculated from the following equations:

(9) $$c_{TS\left(x_{TS}=0\right)}^{\prime }=\frac{K_{DPPC-TS}\left(S_{DPPC}-S_{DPPC-TS}\right)-S_{DPPC}S_{TS}}{S_{DPPC}^{2}(\;S_{DPPC}+K_{DPPC-TS})}$$

(10) $$c_{TS\left(x_{TS}=1\right)}^{\prime }=\frac{-K_{DPPC-TS}\left(S_{TS}-S_{DPPC-TS}\right)-S_{DPPC}S_{TS}}{S_{TS}^{2}(K_{DPPC-TS}-S_{TS})}$$

Equations (9) and (10) may be used for verification of slopes obtained either from theory or by experiment. Agreement between the slopes indicates that the method of calculating $K_{DPPC-TS}$ and $S_{DPPC-TS}$ is justified. 

The complex formation energy of the 1,2-Dipalmitoyl-sn-glycero-3-phosphocholine–triterpene sapogenin system was calculated from Equation (11):

(11) $$-\log K=\frac{\Delta G^{0}}{2.3RT}$$

where K (m^2^ mol^−1^) is the stability constant of 1,2-dipalmitoyl-sn-glycero-3-phosphocholine–triterpene sapogenin complex; $\Delta G^{0}$ (J mol^−1^) is the 1,2-dipalmitoyl-sn-glycero-3-phosphocholine–triterpene sapogenin complex formation energy; R (J mol^−1^K^−1^) is the gas constant; T(K) is the temperature.

## 2. Results and Discussion

The article presents measurements of surface pressure as a function of the monolayer surface area in DPPC-TS systems obtained by the Langmuir method. First, the measurements and analysis of the π-A isotherms were carried out for pure compounds (DPPC, OA, and AA), and then for the lipid mixtures with the mentioned acids. At the same time, in real time, the BAM visualization of the created monolayers was carried out, which provided us with information about the phase transitions that the monolayers underwent during compression. The formation of 1:1 complexes has been demonstrated in DPPC-OA and DPPC-AA systems at the air/water interface. Moreover, the system of equations was determined (see Section 1.1, Equations (1)–(11)), which allowed us to describe the necessary parameters of the obtained complexes. In addition, the values of compressibility moduli and excess free enthalpy of mixing (∆G^exc^) versus monolayer composition for the DPPC-TS system on water subphase were examined. For this purpose, the Equations (12) and (13) for the compressibility modulus and excess mixing enthalpy were used, respectively:

(12) $$C_{S}^{-1}=-A{\left(\frac{d\pi }{dA}\right)}_{T}$$

(13) $$∆G^{exc}=N_{A}\int_{0}^{\pi }A^{exc}\;d\;\pi$$

### 2.1. Surface Pressure—Area Isotherms with BAM Visualization 

**Figure 2:**
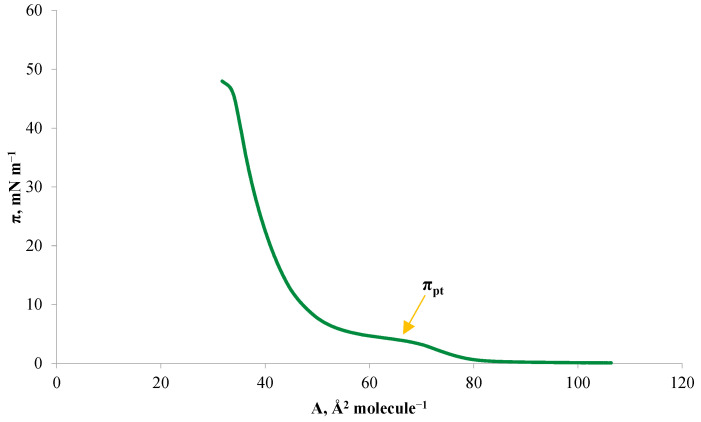
Isotherm of 1,2-Dipalmitoyl-sn-glycero-3-phosphocholine (DPPC).

Figure 2 shows a pure DPPC monolayer. The π-A isotherm shows a typical plateau region of the coexistence of the expanded liquid phase and the condensed liquid phase (LE-LC) at surface pressures from ~4 to 6 mNm^−1^. This phase transition pressure (π_pt_) value is within the range of pressures shown previously in the literature [32,33]. The surface area of a single molecule in the DPPC monolayer was 46 Å^2^molecule^−1^. The literature value of the surface area of a phosphatidylcholine molecule has a wide range and is 45.5–96 Å^2^ molecule^−1^ [30,34]. The experimentally obtained surface area value is within the given literature range.

**Figure 3:**
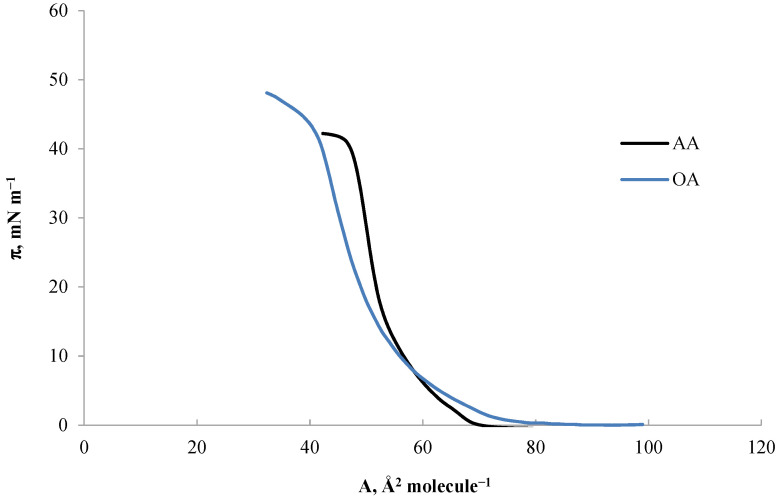
Isotherms of OA and AA.

Figure 3 shows the π-A isotherms for the two tested triterpene sapogenins—oleanolic acid (OA) and asiatic acid (AA). A relatively mild course characterizes the OA and AA isotherm until the surface pressure reaches ~10 mN m^−1^. After exceeding this value, the surface pressure increases rapidly up to the collapse pressure for OA and AA, which is~42.20 and ~40.20 mN m^−1^, respectively.

**Figure 4:**
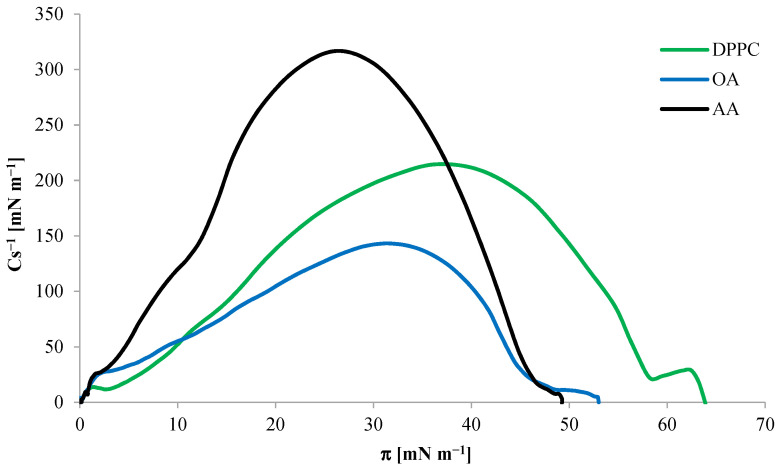
Dependence of the compressibility coefficient Cs^−1^ on the surface pressure πin DPPC, OA, and AA monolayers at 20 °C.

Based on pure monolayers of the tested substances, from Equation (12), calculations were made of the compressibility coefficient Cs^−1^ as a function of the surface pressure π during the monolayer compression, and these relationships are presented in Figure 4.

As shown in Figure 4, the maximum compressibility coefficient Cs^−1^_max_ for DPPC was 209 mN m^−1^, indicating the liquid condensed state of the monolayer. For OA and AA, Cs^−1^_max_ was 143 mN m^−1^ and 317 mN m^−1^, respectively. The value of the compressibility modulus for OA indicates that, at the moment of maximum packing, the monolayer reached the LC state, as in the case of DPPC, and the value of the maximum compressibility modulus of the AA monolayer indicates the presence of a solid state.

The formation of a pure monolayer composed of OA (Figure 3) started at a pressure of ~0.28 mN m^−1^, which corresponds to an A_lift-off_ area of approximately 80 Å^2^ molecule^−1^. The boundary area per single molecule in the OA monolayer (A_lim_) was 54 Å^2^ molecule^−1^, similar to those presented in the literature [35].

**Figure 5:**
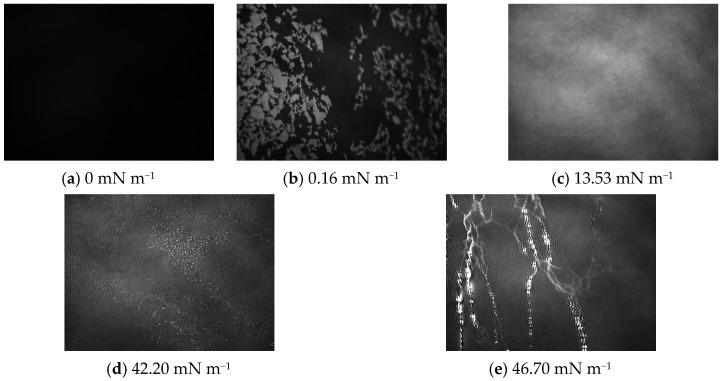
Brewster angle microscope (BAM) images of the OA monolayer. The images were taken during compression at a constant temperature of 20 °C in a field of view of 3.6 × 4.0 mm. A black glass plate immersed in the subphase absorbed the refracted beam. The image resolution was approximately 6 μmpixel^−1^.

At the same time, while compressing the monolayer, the BAM images presented in Figure 5 were recorded.

Based on the π-A isotherm for oleanolic acid (Figure 3) and BAM images (Figure 5), which were recorded during the compression of the pure OA monolayer, a description of its phase transitions was made. In the initial phase of the formation of the OA monolayer, when the curve takes the form of an almost parallel line to the *x*-axis, the molecule surface in the monolayer is large (A > 80 Å^2^ molecule^−1^) and the surface pressure is low (~0 mN m^−1^), the BAM image is homogeneous with very low reflectance, and the image is completely dark (Figure 5a), which indicates the presence of a monolayer in the gaseous state (G). In the further part of the curve, an increase in surface pressure (~1 mN m^−1^) is observed with a simultaneous decrease in the molecular surface area, which indicates a gradual transition of the monolayer to the LE state (Figure 5b). The BAM image is observed as a bright, irregular submerged area in the darker phase (subphase image). At a surface pressure of approximately 10 mN m^−1^, when the chains of molecules in the monolayer gradually organize, we observe the expanded liquid phase LE (Figure 5c) already forming a homogeneous area. In the condensed liquid phase (Cs^−1^ = 50–250 mN m^−1^), the surface pressure increases rapidly from ~10 mN m^−1^ to the collapse pressure (~42.20 mN m^−1^), while showing small, round, bright points (Figure 5d). This image shows domains with different brightness, which is related to the orientation of the molecules in the monolayer and their packing state. The brighter the areas are, the more packed the monolayer is. After exceeding the collapse pressure, three-dimensional structures are observed, which, for OA, take the form of long, interconnected, bright structures (Figure 5e).

**Figure 6:**
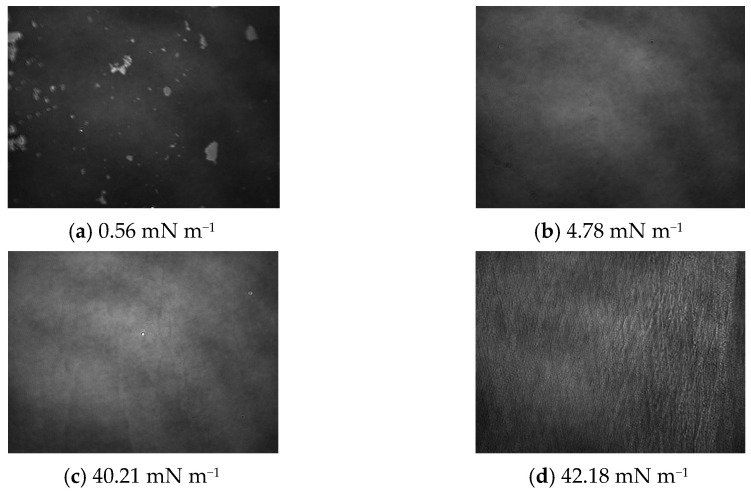
Brewster angle microscope (BAM) images of the AA monolayer. The images were taken during compression at a constant temperature of 20 °C in a field of view of 3.6 × 4.0 mm. A black glass plate immersed in the subphase absorbed the refracted beam. The image resolution was approximately 6 μmpixel^−1^.

Through the analysis of the AA isotherm (Figure 3) and BAM images (Figure 6), it could be concluded that the A_lift-off_ value was approximately 69 Å^2^ molecule^−1^ at a pressure of ~0.29 mN m^−1^, which also illustrates the gradual transition of the monolayer to LE state (Figure 6a). Further compression, unlike OA, already at a surface pressure of ~4.70 mN m^−1^causedthe created monolayer to enter the LC state (Figure 6b), where the BAM image appears as a homogeneous area intermediate between black and light gray, without separated, visible structures. From the surface pressure of ~17 mN m^−1^ to the collapse pressure of the monolayer, a condensed phase of the monolayer was observed. The obtained average value of the surface area per single molecule in the AA monolayer was 56 Å^2^ at a collapse pressure of ~40 mN m^−1^(Figure 6c). The monolayer molecules formed delicate, bright, and long domains with these parameters. Further compression caused the AA domains to take on the structure of long, regularly interconnected ‘fibers’, visible as a ‘sweater’ structure (Figure 6d).

Comparing both isotherms for OA and AA acids, presented earlier (Figure 3), we can notice a similarity in the course of changes in surface pressure during compression of both monolayers. Both the surface pressures near the surface of A_lim_ and the collapse pressures are similar. At the same time, for both compounds, a sharp jump in the curve is observed from the surface pressure of about 10 mN m^−1^. However, despite the similarity in the course of isotherms, the AA curve is distinguished by a greater inclination angle of the *x*-axis after exceeding the surface pressure mentioned above and a higher maximum compressibility coefficient. This proves that the AA monolayer reaches the LC phase state faster. Its structure is more rigid than the structure of the OA monolayer, which also shows the dependence of the compressibility coefficient on the surface pressure In monolayers (Figure 4). The maximum compressibility coefficient indicates the LC state of the OA monolayer and the condensed state of the AA monolayer. The parameters of the surface pressure at the moment of collapse of the monolayer and the molecular boundary surface in the DPPC monolayer differ from the values obtained for the tested triterpene sapogenins. Table 1 shows detailed summary data for DPPC, OA, and AA monolayers.

### 2.2. Mixed Monolayer Experiment and Complex Formation

The interactions between DPPC and TS can be explained by the formation of complexes in these systems. 1,2-Dipalmitoyl-sn-glycero-3-phosphocholine was modified with oleanolic acid and asiatic acid. A series of mixtures with various concentrations of tested compounds was prepared, and then π-A isotherms were registered.

#### 2.2.1. DPPC–OA Mixed Monolayers

**Figure 7:**
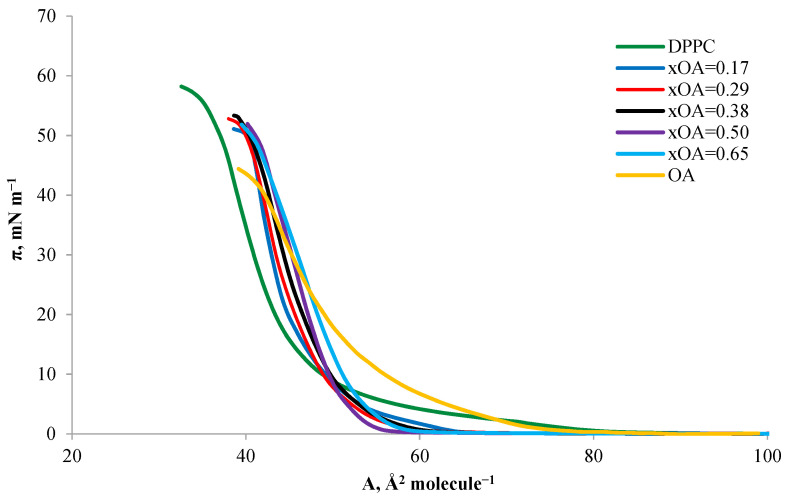
Surface pressure–area (π-A) isotherms of mixed DPPC-OA monolayers.

Measurements of π-A isotherms were carried out for the DPPC-OA system mixtures in the mole fraction of OA, as shown in Figure 7. The obtained curves showed that the surface area occupied by a single molecule in the monolayers increased with increasing oleanolic acid content in the DPPC-OA mixture.

A common feature of all DPPC–OA mixed isotherms was a steeper slope than the isotherms of pure DPPC and OA components. This may indicate a stiffer structure of the resulting mixed monolayers. As the OA content in DPPC–OA mixed monolayers increased, the surface area of a single molecule increased. The collapse pressure varied from the highest for DPPC to the lowest for OA, which may indicate a faster achievement of a more ordered monolayer state with increasing OA content in the mixed monolayers.

**Figure 8:**
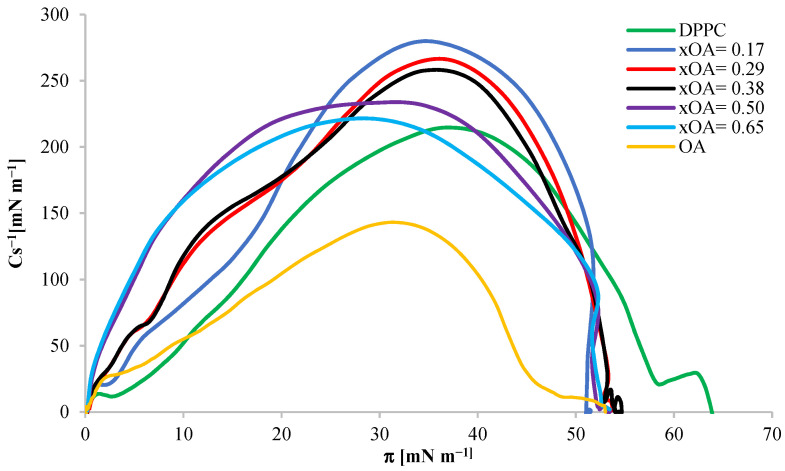
Dependence of the compressibility coefficient Cs^−1^ on the surface pressure in mixed monolayers for the DPPC-OA system with different mole fraction ratios.

Based on the obtained DPPC–OA mixed monolayers from Equation (12), the dependences of the compressibility coefficients as a function of the surface pressure of the tested monolayers were determined, depending on their composition. The Cs^−1^ = f(x) curves are shown in Figure 8.

The curves presented in the graph show that the compressibility coefficients for all mixed monolayers reach higher values than those for monolayers composed of pure ingredients. The highest values of the compressibility coefficient Cs^−1^_max_, presented in the Cs^−1^ = f(π) relationship chart, are achieved by monolayers of mixtures with the mole fraction x_OA_ = 0.17–0.38 (Cs^−1^_max_ > 250 mN m^−1^), which proves that the formation of monolayers reaches the condensed state. The remaining mixtures with mole fractions x_OA_ = 0.5–0.65 form liquid monolayers (50 mN m^−1^ < Cs^−1^_max_ < 250 mN m^−1^). Moreover, a decrease in the value of the compressibility modulus in mixed monolayers is observed with the increasing OA content in the model monolayer membrane composed of DPPC.

**Figure 9:**
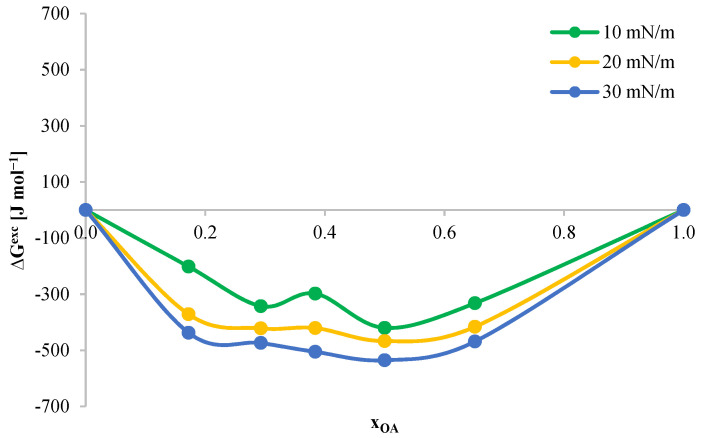
Dependence of the change in excess free enthalpy of mixing (ΔG^exc^) on the mole fraction of OA in mixed monolayers for the DPPC-OA system.

A quantitative analysis of the interactions between molecules in mixed monolayers was also performed based on calculations of changes in the excess free enthalpy of mixing (ΔG^exc^) as a function of the monolayer composition, based on Equation (13). Changes in the value of the excess free enthalpy of mixing ΔG^exc^ indicate the thermodynamic stability of the system. Changes in the excess free enthalpy of mixing ΔG^exc^ were determined at selected surface pressure values, i.e., 10 mN m^−1^, 20 mN m^−1^, and 30 mN m^−1^. The obtained ΔG^exc^ results as a function of mole fractions of the OA are presented in Figure 9.

Negative values of the function ΔG^exc^ = f(x_OA_) in the range of tested surface pressures (10–30 mN m^−1^) indicate the existence of attractive molecular interactions between DPPC and OA molecules. The created monolayers are characterized by high stability over the entire concentration range. Additionally, the stability of the tested system depends on the surface pressure and increases with its increase. The minimum values during the ΔG^exc^(x_OA_) function, illustrating the strongest interactions between monolayer components, may indicate the formation of DPPC–OA surface complexes. Previous studies suggest the possibility of forming surface complexes between components in systems with 1:1 stoichiometry [30,36]. In the above graph, the minimum ΔG^exc^ values are achieved for mixtures with x_OA_ = 0.5, which also allows us to conclude the formation of such a complex in the DPPC–OA system in a mixture with a stoichiometry of 1:1.

**Figure 10:**
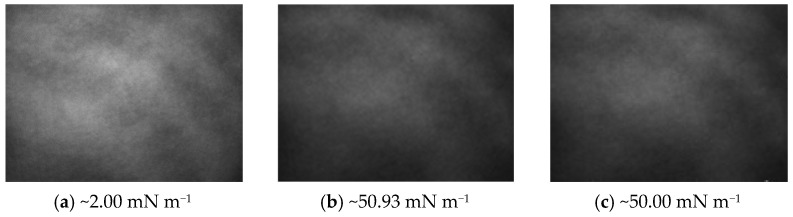
Brewster angle microscopy (BAM) images of mixed DPPC-OA monolayers (1:1 stoichiometric ratio). The photos were taken during compression at a constant temperature of 20 °C, with a field of view of 3.6 × 4.0 mm. A black glass plate immersed in the subphase absorbed the refracted beam. The image resolution was approximately 6 μmpixel^−1^.

Assuming the formation of complexes with a 1:1 stoichiometry between the tested compounds, BAM photos were visualized and described in DPPC–OA mixed monolayers, and the recorded microscopic images for the DPPC–OA system are presented in Figure 10.

For the DPPC–OA monolayer with 1:1 stoichiometry, a rapid increase in the compressibility coefficient was noticed in the initial phase of its formation (Figure 8), which indicates that the monolayer achieved the LC state already at a surface pressure of about 2 mN m^−1^ (Figure 10a). A gray picture with no extracted structures is observed in the BAM image. BAM images at the monolayer collapse pressure and after exceeding π_coll_ (Figure 10b,c) still do not show any isolated structures, which may indicate very good miscibility of this system with a stoichiometric ratio of 1:1.

#### 2.2.2. DPPC–AA Mixed Monolayers

**Figure 11:**
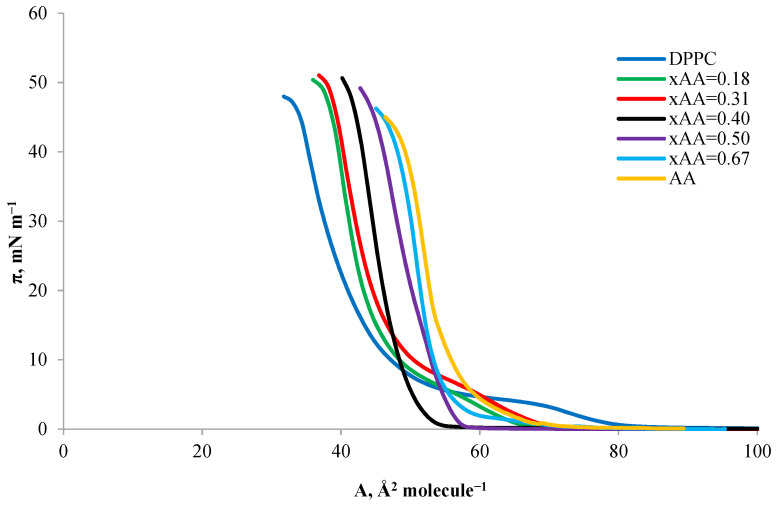
Surface pressure–area (π-A) isotherms of mixed DPPC-AA monolayers.

As in the DPPC–OA system, in the DPPC–AA system, the graph in Figure 11 shows a shift in the surface area of a single molecule towards higher values, with an increase in the AA content in mixed monolayers. The collapse pressure increases from the DPPC isotherm to the DPPC–AA isotherm with a mole fraction x_AA_ = 0.4. Then, it decreases to the lowest value for a single-component monolayer composed of AA. This allows us to conclude that the high AA content in the DPPC–AA mixed monolayer stiffens its structure, which is visible as the monolayer reaches π_coll_ more quickly.

**Figure 12:**
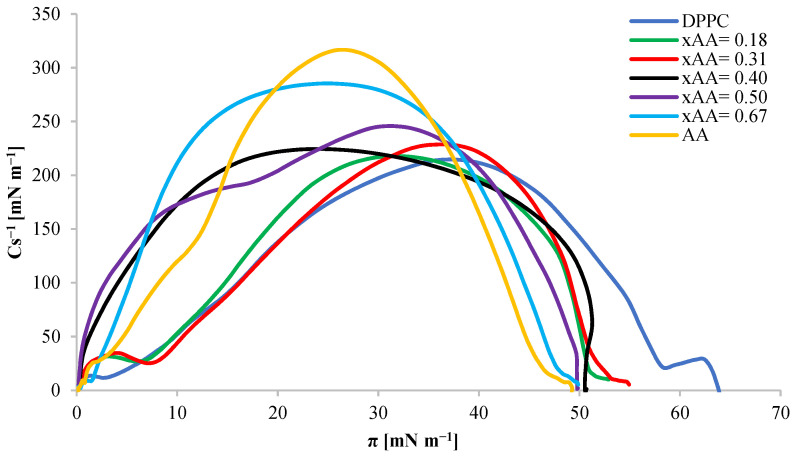
Dependence of the compressibility coefficient Cs^−1^ on the surface pressure in mixed monolayers for the DPPC-AA system with different mole fraction ratios.

For each of the DPPC–AA mixed monolayers, compressibility coefficients were determined from Equation (12), and the obtained curves are shown in Figure 12.

The compressibility coefficients for mixed monolayers have values between the compressibility coefficients for monolayers composed of pure components. The highest Cs^−1^_max_ values presented in the Cs^−1^ = f(π) relationship chart are achieved by monolayers of mixtures with the mole fraction x_AA_ = 1 and 0.67 (Cs^−1^_max_ > 250 mN m^−1^), which proves that these monolayers have achieved condensed state. Other mixtures with mole fractions x_AA_ = 0.18, 0.31, 0.40, and 0.50 form monolayers of a condensed liquid nature (50 mN m^−1^ < Cs^−1^_max_ < 250 mN m^−1^).

**Figure 13:**
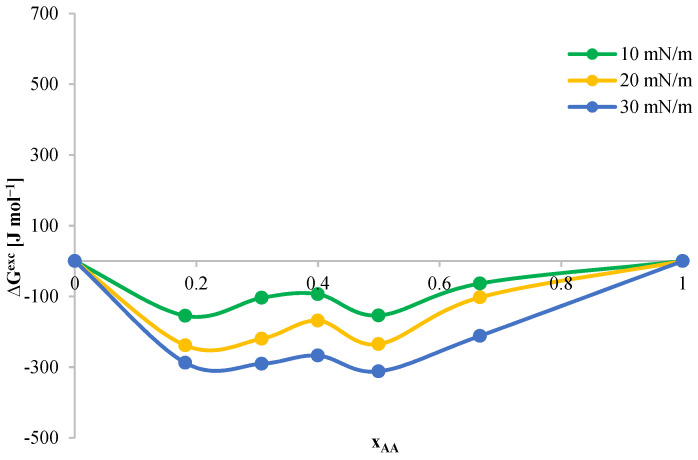
Dependence of the change in excess free enthalpy of mixing (ΔG^exc^) on the mole fraction of AA in mixed monolayers for the DPPC-AA system.

The interactions between components in DPPC–AA mixed monolayers were quantitatively described. Changes in the excess free enthalpy of mixing ΔG^exc^ were again calculated (Equation (13)). The obtained ΔG^exc^ results as a function of mole fractions of the tested compound are presented in Figure 13.

The function ΔG^exc^ = f(x_AA_) takes on negative values in the entire range of tested surface pressures. This proves the existence of attractive intermolecular interactions between DPPC and AA. The created monolayers are characterized by high stability over the entire concentration range. Additionally, the stability of the tested system depends on the surface pressure and increases with its increase. The minimum values in the course of the ΔG^exc^(x_AA_) function are observed at x_AA_ equal to 0.5, which indicates the formation of a complex in a mixture with this molar composition.

**Figure 14:**
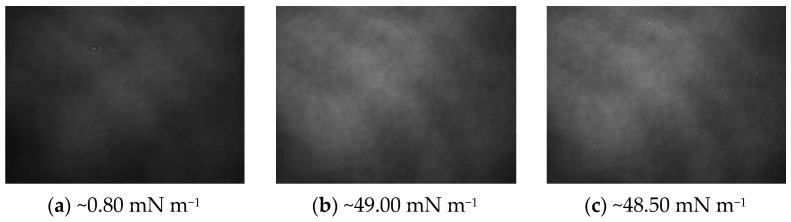
Brewster angle microscopy (BAM) images of mixed DPPC-AA monolayers (1:1 stoichiometric ratio). The photos were taken during compression at a constant temperature of 20 °C with a field of view of 3.6 × 4.0 mm. A black glass plate immersed in the subphase absorbed the refracted beam. The image resolution was approximately 6 μm pixel^−1^.

As in the previous case, also for the DPPC–AA system, the assumption of complexes forming in mixed monolayers with a stoichiometric ratio of 1:1 was considered. BAM images were also recorded and annotated for these mixtures; the recorded microscopic images are shown in Figure 14.

In the initial phase of creating a mixed monolayer with a mole fraction x_AA_ = 0.5, an increase in the compressibility coefficient is observed very quickly (Figure 12). Already at a pressure of ~0.80 mN m^−1^, it is possible to observe the liquid phase condensed in the monolayer, which, in the BAM image (Figure 14a) appears as a homogeneous image without visible isolated structures. After organizing the monolayer at a pressure of π_coll_ (Figure 14b), it is still not noticeable there are no domains. As in the case of the DPPC–OA system, this may indicate good miscibility of the compounds in this stoichiometric ratio. After exceeding the pressure π_coll_ of the monolayer, no distinct structures are noticed (Figure 14c).

#### 2.2.3. Complex Formation

**Figure 15:**
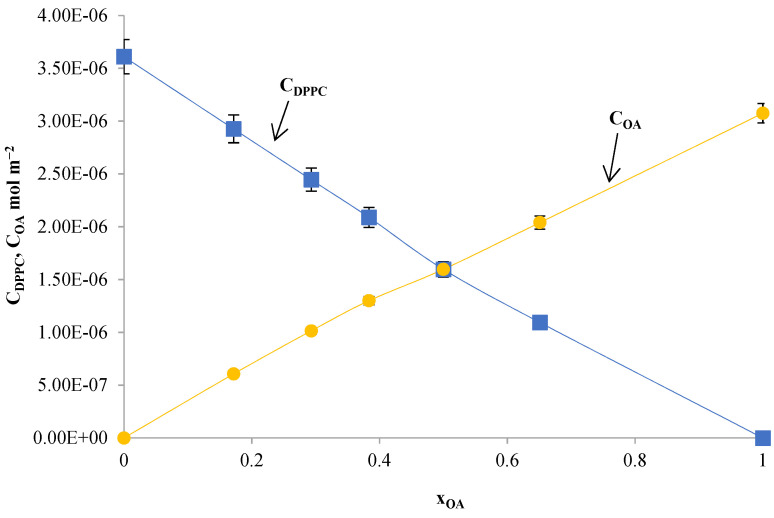
Dependence of the total surface concentration of DPPC (c_DPPC_) and oleanolic acid (c_OA_) vs. the mole fraction of oleanolic acid. In the graph, the points represent the experimental values, and the theoretical values are shown by the curve.

**Figure 16:**
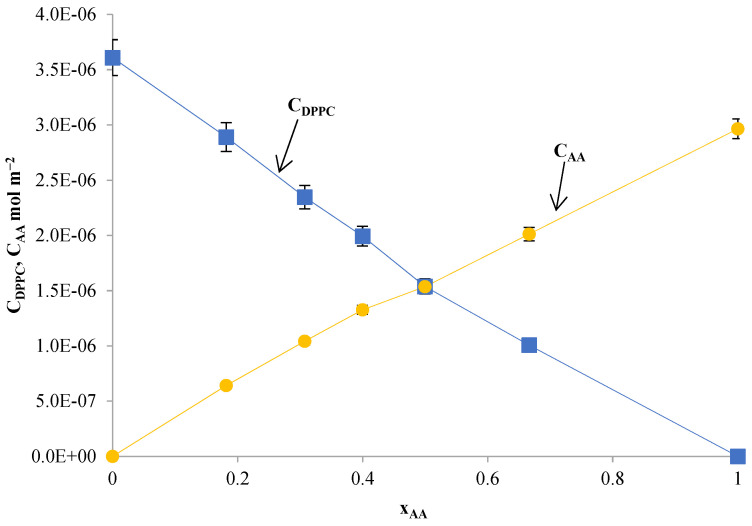
Dependence of the total surface concentration of DPPC (c_DPPC_) and asiatic acid (c_AA_) vs. the mole fraction of asiatic acid. In the graph, the points represent the experimental values, and the theoretical values are shown by the curve.

It is assumed that the complexes DPPC-OA and DPPC-AA are formed in the ratio 1:1 (Equations (1)–(5)) and are characterized by the stability constants K_DPPC–TS_ (Equation (5)). Figure 15 and Figure 16 show the total surface concentration of DPPC-TS as a function of the mole fraction of TS. As can be seen, the function c_DPPC_ = f (x_DPPC_) is practically linear for x_DPPC_ > 0.5, confirming the monolayer’s condensed nature. 

It was established that DPPC and OA form a 1:1 DPPC-OA complex in the monolayer. One of the parameters characterizing this complex is the K_DPPC-OA_ stability constant, equal to 5.97 × 10^5^ m^2^ mol^−1^; this value was calculated as shown in Equation (7). The area of the S_DPPC-OA_ complex was 5.35 × 10^5^ m^2^ mol^−1^ (88.79 ± 0.9 Å^2^ of molecule^−1^), which was calculated as shown in Equation (8). Complex formation energy (Gibbs free energy) for the DPPC-OA complex, calculated from Equation (11), was equal to −32.58 ± 0.33 kJ mol^−1^.

The total surface concentrations of DPPC (c_DPPC_) and AA (c_AA_) as a function of the mole fraction of AA are presented in Figure 16. The parameters of the complex, such as the stability constant (K_DPPC-AA_ = 1.03 × 10^6^ m^2^ mol^−1^) and the area per complex DPPC-AA (S_DPPC-AA_ = 4.69 × 10^5^ m^2^ mol^−1^, 77.93 ± 0.8 Å^2^ particle^- 1^),were calculated theoretically as before from the Equations (7) and (8).

The value of the complex formation energy (Gibbs free energy) for the DPPC-AA complex was −33.91 ± 0.34 kJ mol^- 1^. All physicochemical parameters characterizing the resulting DPPC-OA and DPPC-AA complexes in the monolayers are presented in Table 2.

The DPPC–TS stability constant was approximately 5.97 × 10^5^–1.03 × 10^6^ m^2^ mol^−1^, whereas the stability constant of the lipid–stearic sapogenine complex was 3.00 × 10^5^–6.5 × 10^5^ m^2^ mol^−1^ [31,37]. These values are relatively high and allow one to argue that the discussed systems have a similar structure. The relatively high stability of DPPC–TS provides additional evidence for the prevalence of the 1:1 complex in mixed phospholipid–sapogenin monolayers.

Knowledge of this thermodynamic function provides information concerning the nature and type of bonding in the tested systems and groups taking part in the complex forming reactions, for which there are many areas of application in chemistry, biology, and medicine. 

The complex formation energy value for the DPPC–OA and DPPC–AA systems was approximately −(32–33) kJ mol^−1^. The values presented above are close to those determined earlier for similar systems (e.g., PC–diosgenin or PC–flavonoid systems), which were −(30–40) kJ mol^−1^ [31,38].

## 3. Materials and Methods

### 3.1. Materials

Semisynthetic DPPC (1,2-Dipalmitoyl-sn-glycero-3-phosphocholine, ≥99%), oleanolic acid(≥97%, OA), and asiatic acid (97%, AA) were purchased from Sigma-Aldrich (Schnelldorf, Niemcy) and were used in the research without any further purification. The molecular weight of the compounds was 752.08, 488.70, and 456.70 g mol^−1^, respectively. All study substances were dissolved in chloroform 99.8% (HPLC, Chempur, Poland) in a concentration of 1 mg mL^−1^. A 5% addition of methanol (99.8% PURE P.A.–BASIC, POCH, Poland) was used to dissolve the AA completely.

### 3.2. Methods

#### 3.2.1. Langmuir Monolayer Method

The surface pressure–molecular area (π-A) measurements were carried out at the air/water interface at 20 °C by using a Langmuir trough (KSV NIMA, Espoo, Finland) combined with a Brewster angle microscope (ultraBAM from Accurion, Germany). The measuring block consisted of the trough (the large type, KN 1006) with an area of 841 cm^2^ made of Teflon (PTFE) and two symmetrical barriers made of Delrin (polyoxymethylene). The trough was filled with high-purity deionized water (20 °C ± 1 °C, Hydrolab system, Straszyn, Poland) as a subphase for all measurements. The monolayers were prepared by spreading the solutions over the subphase with a Hamilton microsyringe (precise ±2 μL). Both lipids and tested sapogenins were dissolved in chloroform, and the concentration of the solutions was 1 mg mL^−1^. To better dissolve asiatic acid, the 5% addition of methanol was used. The very sensitive platinum Wilhelmy plate was used as a surface pressure sensor. The surface pressure, temperature, and symmetrical barrier position changes were controlled with a particular detection unit. Graphs of the surface pressure dependence as a function of the surface area of molecules (π-A) in monolayers were obtained directly from the KSV NIMA computer program. Every isotherm was repeated at least three times to ensure reproducibility of the curves to ±1 Å^2^molecule^−1^.

#### 3.2.2. Brewster Angle Microscopy Method 

The floating monolayer texture was visualized using a Brewster angle microscope (ultraBAM, Accurion GmbH, Goettingen, Germany)combined with the Langmuir system (KSV NIMA, Finland). The method was used to obtain images of created films in real time. The BAM system contains a 50 mW He–Ne laser emitting p-polarized light with a wavelength of 658 nm and a CCD camera with a resolution (1360 × 1024 pixels). The whole device was located on a unique antivibration table and was surrounded by a plexiglass enclosure to eliminate the factors from the external environment. The Langmuir monolayers were prepared as described before. The minimum reflection angle for the pure water was set at the Brewster angle (53°). The BAM images were taken with stationary Langmuir barriers at a temperature of 20 °C ± 1 °C.

## 4. Conclusions

The presented data related to the physicochemical properties of phospholipids modified with asiatic and oleanolic acids indicate interactions between the membrane components. Due to a larger number of polar groups in the molecules of the tested sapogenins, their arrangement in the monolayer at the water/air interface is non-uniform, and the monolayer itself shows a heterogeneous structure. According to Brezesinski’s research, the OA molecule will most likely be arranged perpendicularly when the -COOH group is directed toward the aqueous phase. The -COOH group is more polar and more capable of forming hydrogen bonds, so the -OH group aligns itself towards the gas phase [39,40]. On this basis, it can be assumed that the arrangement of the AA molecule in the monolayer will be similar. As mentioned earlier, the pure sapogenin monolayer may be unstable; however, as the results obtained with DPPC show, this system shows better stability.

Wojciechowski et al. [41] studied the adsorption of three triterpene saponins (α-hederin, hederacoside C, and ammonium glycyrrhizate) onto a free water surface and Langmuir monolayers of DPPC and DPPC/cholesterol (10:9 mol/mol). They showed that saponin affects the layers to the highest extent, especially when cholesterol is present. They also studied saponin–lipid complex formation in ethanol–aqueous homogeneous solutions using UV/Vis spectroscopy. The oleanolic-acid-type saponins (α-hederin and hederacoside C) were shown to form complexes with lipids characterized by stability constants in the range (4.0 ± 0.2) × 10^3^–(5.0 ± 0.4) × 10^4^ M^−1^. The stability constants of the formation of complexes between lipid and triterpene sapogenin described in this article are consistent with the results presented by Wojciechowski et al. [41], which confirms the validity of our assumptions regarding the formation of a complex between lipids and triterpene sapogenins in a monolayer.

In conclusion, the tested compounds from the group of triterpene sapogenins form complexes with phosphatidylcholine with a stoichiometry of 1:1 (molar fraction x_DPPC_:x_AA_ = 0.5). Monolayers mixed in this molar ratio are characterized by high stability, and the stability constants of the formed complexes are high, which was supported by experimental data and theoretical calculations. The assumption about the possibility of forming complexes with a 1:1 stoichiometry in the phosphatidylcholine–triterpene sapogenin system, based on previous research results with steroid sapogenins [31,37], turned out to be correct.

## Figures and Tables

**Table 1:** Determined parameters of the tested single-component isotherms DPPC, OA, and AA.

	A_lift-off_ (Å^2^molecule^−1^)	π_coll_ (mN m^−1^)	A_lim_ (Å^2^molecule^−1^)	Cs^−1^_max_ (mN m^−1^)
DPPC	83	48	46	209
OA	80	42	54	143
AA	69	40	56	317

**Table 2:** Physicochemical parameters determined for the tested systems: DPPC-OA and DPPC-AA.

Examined System	Surface Area of Complex (Å^2^molecule^−1^)	Stability Constant (m^2^ mol^−1^)	Complex Formation Energy (kJ mol^−1^)
DPPC-OA	88.79 ± 0.89	5.97 × 10^5^	−32.58 ± 0.33
DPPC-AA	77.93 ±0.78	1.03 × 10^6^	−33.91 ±0.34

## Data Availability

The data presented in this study are available on request from the corresponding author.
